# Lycopene Protects against Hypoxia/Reoxygenation-Induced Apoptosis by Preventing Mitochondrial Dysfunction in Primary Neonatal Mouse Cardiomyocytes

**DOI:** 10.1371/journal.pone.0050778

**Published:** 2012-11-30

**Authors:** Rongchuan Yue, Houxiang Hu, Kai Hang Yiu, Tao Luo, Zhou Zhou, Lei Xu, Shuang Zhang, Ke Li, Zhengping Yu

**Affiliations:** 1 Department of Cardiology, North Sichuan Medical College First Affiliated Hospital, Nanchong, Sichuan, China; 2 Center for Medical Research, North Sichuan Medical College First Affiliated Hospital, Nanchong, Sichuan, China; 3 Department of Medicine, University of Hong Kong, Hong Kong, China; 4 Department of Occupational Health, Third Military Medical University, Chongqing, China; University of Pecs Medical School, Hungary

## Abstract

**Background:**

Hypoxia/reoxygenation(H/R)-induced apoptosis of cardiomyocytes plays an important role in myocardial injury. Lycopene is a potent antioxidant carotenoid that has been shown to have protective properties on cardiovascular system. The aim of the present study is to investigate the potential for lycopene to protect the cardiomyocytes exposed to H/R. Moreover, the effect on mitochondrial function upon lycopene exposure was assessed.

**Methods and Findings:**

Primary cardiomyocytes were isolated from neonatal mouse and established an in vitro model of H/R which resembles ischemia/reperfusion in vivo. The pretreatment of cardiomyocytes with 5 µM lycopene significantly reduced the extent of apoptosis detected by TUNEL assays. To further study the mechanism underlying the benefits of lycopene, interactions between lycopene and the process of mitochondria-mediated apoptosis were examined. Lycopene pretreatment of cardiomyocytes suppressed the activation of the mitochondrial permeability transition pore (mPTP) by reducing the intracellular reactive oxygen species (ROS) levels and inhibiting the increase of malondialdehyde (MDA) levels caused by H/R. Moreover, the loss of mitochondrial membrane potential, a decline in cellular ATP levels, a reduction in the amount of cytochrome c translocated to the cytoplasm and caspase-3 activation were observed in lycopene-treated cultures.

**Conclusion:**

The present results suggested that lycopene possesses great pharmacological potential in protecting against H/R-induced apoptosis. Importantly, the protective effects of lycopene may be attributed to its roles in improving mitochondrial function in H/R-treated cardiomyocytes.

## Introduction

Myocardial infarction is the leading cause of premature death in the United States and many developed countries [1], [2]. Early reperfusion after myocardial infarction is essential for reestablishing the blood flow in order to prevent the myocardium from further damage. Nevertheless, ischemia and reperfusion (I/R)-injury is inevitable, which limits the myocardial salvage. Although the exact mechanism is uncertain, several hypotheses have been proposed to describe the pathogenesis of myocardial I/R-injury: including oxygen radical hypothesis, calcium overload hypothesis and inflammatory hypothesis [3]. As a result, the exploration for a new intrinsic or exogenous method and potential therapeutic agents that aims at reducing the I/R-injury has become an area of intensive research.

Lycopene, a carotenoid compound, found naturally in tomato and other fruits like papaya, pink guava and watermelon has long been known for its health-promoting ability [4]. Among naturally occurring carotenoids, lycopene has the strongest ability to scavenge free radicals; being 10-fold, 47-fold and 100-fold more effective at quenching singlet oxygen than α-tocopherol, β-carotene and vitamin E respectively [5], [6]. Thus, lycopene treatment may represent a new therapeutic strategy in treating ROS-related pathophysiological damage.

With the strong antioxidant property, lycopene has been shown to have the ability to reduce the risk of cancers, such as breast, prostate and pancreas cancer [7]. It also has protective effect in various chronic conditions like cardiovascular disease (CVD), coronary heart disease (CHD) and atherosclerosis [8], [9], [10]. In fact, higher plasma lycopene concentration is associated with lower risk of CVD in women which is mediated through the regulation of cholesterol metabolism [11].

Nevertheless, little is known about the role of lycopene in cardioprotection and the underlying protective mechanisms during I/R. Besides oxidative stress and apoptosis [12], mitochondria dysfunction [13], [14] has been shown to exert a key role in myocardial reperfusion injury. Therefore, the aims of the study were to ascertain the protective effects of lycopene on H/R-induced apoptosis and to assess whether the mitochondrial dysfunction was involved in these effects.

In this study, we isolated primary cardiomyocytes from neonatal mouse and established an in vitro model of hypoxia/reoxygenation (H/R) which resembles I/R in vivo. Then we investigated the effects of lycopene on H/R injury and discovered that lycopene protects against H/R i-induced apoptosis by improving mitochondrial function.

## Materials and Methods

### Primary Cardiomyocyte Culture

Cardiomyocytes were prepared from Neonatal C57BL/6 mice (Animal Center of the Third Military Medical University, Chongqing, China). This study was carried out strictly in accordance with the recommendations in the Guide for the Care and Use of Laboratory Animals of the National Institutes of Health. The protocol was approved by the Committee on the Ethics of Animal Experiments of the Third Military Medical University (Permit Number: SYXK(CQ)2007-002). The neonatal mice (1–3 day old) were disinfected with 70% ethanol and then killed by decapitation. The chest was opened and the heart was rapidly removed and placed in the cold CBFHH solution that contains in mM: NaCl 145, D-Glucose 10, KCl 5.4, MgCl_2_·6H_2_O 1.2, Na-pyruvate 2, HEPES 10. These hearts were minced with a fine forceps and collected. These tissue fragments were digested by 0.1% trypsin (Hyclone, SH30042.01) for 10 min in a tube in 37°C water bath. After that, the cell suspension was centrifuged (300 g for 8 min at 4°C). The supernatant was then removed and the cell pellet was resuspended in M199 medium (HyClone SH30253.01B) supplemented with 10% fetal bovine serum (HyClone, Logan, UT, USA), 0.5 mM L-Glutamine (Japan, B212), and 1% (v/v) streptomycin/penicillin (Beyotime, C0222). These steps were repeated until the tissue fragments had disappeared. The dissociated cells were replated in a culture flask at 37°C for 1 h to enrich the culture with cardiomyocytes. The nonadherent cardiomyocytes were collected and cells were counted by TC10™ Automated cell counter (BIO-RAD Singapore, 506BR3124) and then they were plated onto gelatin-coated well plates. The cells were cultured in M199 medium supplemented with 10% fetal bovine serum, 0.5 mM L-Glutamine, 0.1 mM Bromodeoxyuridine (Brdu) (Sigma, B5002) which was used to prevent proliferation of cadiac fibroblast, and 1% (v/v) streptomycin/penicillin in a 5% CO_2_-humidified atmosphere at 37°C. The growth medium was changed on the following day with a medium that did not contain Brdu.

### Hypoxia/reoxygenation Treatment to Cardiomyocytes

Hypoxia/reoxygenation model was established according to the methods previously described [15], [16], [17], [18], [19], [20], [21] with some modifications. After 48–72 h, when the neonatal cardiomyocytes of mice were cultured to 70% confluence in appropriate culture dishes, they were pre-starved using M199 medium without fetal bovine serum for 12 h. Then hypoxia was induced by replacing the initial culture medium with DMEM without glucose and serum, which was preflushed with a gas mixture (95% N_2_ and 5%CO_2_) for 15 min. Then cardiomyocytes were placed in a modular incubator chamber (Billups Rothenberg, Inc., Del Mar, CA) and were flushed with the gas mixture of 5% CO_2_–95% N_2_ for 60 min. After that, the sealed chamber was placed into a 37°C incubator. After hypoxia incubation, the cells were provided with fresh normal medium and then restored to 95% air and 5% CO_2_ for reoxygenation. For lycopene treatment, lycopene (Sigma–Aldrich) was dissolved in a tetrahydrofuran (THF) solution containing 0.025% butylated hydroxytoluene (BHT) to inhibit formation of peroxides. This stock solution was prepared with minimal exposure to air and light and stored at −70°C. 4 h prior to the H/R treatment, THF-lycopene aliquots from the stock solution were added to the culture medium to the indicated final concentration. The THF was added in control and H/R groups. The amount of THF Vehicle in the culture medium was 0.05% (V/V), a concentration that did not affect the assays as evidenced by comparisons with vehicle-free control medium.

### Viability Assay

Cell viability was assessed with the Trypan blue staining viability test as described previously [22], [23], [24] with some modifications. Briefly, the culture medium was removed and transferred to containers. The cells were digested by 0.25% Trypsin (5 min at 37°C). Then, the removed medium was given back to each well to terminate the digestion and the cell suspensions were centrifuged at 300 g for 15 min at 4°C. The pellets were resuspended in 0.4% Trypan blue solution (Sigma) and incubated for 5 min at 37°C. Both the stained dead cells and the unstained viable cells were counted by cell counter. The cell viability was quantified as the percent of unstained cells over all cells.

### TUNEL Assay for Apoptosis

According to our previous experiments [25], [26], the terminal deoxynucleoitidyl transferase-mediated biotinylated UTP nick end labeling (TUNEL) assay was performed using an in situ cell death detection kit (Roche, USA). The cardiomyocytes on coverslips were rinsed in phosphate-buffered saline (PBS), pH7.4 and subsequently fixed for 60 min in 4% paraformaldehyde, pH 7.4, at room temperature. After being rinsed twice in PBS, the cardiomyocytes were permeabilized with 0.1% TritonX-100 in 0.1% sodium citrate for 10 min at room temperature. The cardiomyocytes were rinsed two times in PBS again and then apoptotic cells were detected by TUNEL staining following the manufacturer’s instructions. At last, cardiomyocytes were counterstained with Hoechst33342 (5 min, at room temperature) (Beyotime, China). TUNEL-positive nuclei were counted in four nonoverlapping fields per coverslip, and then were converted to percentage by comparing TUNEL-positive counts with the total cell nuclei determined by Hoechst33342 counterstaining. Assay was performed in a blinded manner and the experiment was repeated for three times.

### Assay of Intracellular Reactive Oxygen Species (ROS) and Malondialdehyde (MDA) Levels

The determination of intracellular oxidant production was based on the oxidation of 2′, 7′-dichloroﬂuoresce in diacetate (DCFH-DA) (Beyotime, China). Cardiomyocytes were seeded into a 96-plate at a density of 1×10^5^/well. Following the treatment of H/R, cells were incubated with DCFH-DA at 37°C for 20 min. The ﬂuorescence was read at 485 nm for excitation and 530 nm for emission with an Infinite™ M200 Microplate Reader (Tecan, Männedorf, Switzerland). Then, cells were counted in each group in five nonoverlapping fields randomly to normalize the fluorescence intensity. The amount of emitted fluorescence was correlated with the quantity of ROS in the cell. The experiment was repeated for three times, and cellular ﬂuorescence intensity was expressed as the fold change to the control group.

The intracellular MDA levels were measured using a Lipid Peroxidation MDA Assay Kit (Beyotime, China) following the manufacturer's instructions. First, 1×10^6^ cardiomyocytes were seeded in a 6-well plate. After H/R treatment, cardiomyocytes were collected and lysed by cell lysis buffer and centrifuged at 10,000 g for 15 min. The supernatants were reacted with the thiobarbituric acid (TBA), and the reaction products were measured spectrophotometrically at 535 nm. The experiment was repeated for three times, and the MDA levels were expressed as nmol/mg protein.

### Measurement of Mitochondrial Membrane Potential

The fluorescent, lipophilic and cationic probe, JC-1 (Beyotime, China), was employed to measure the mitochondrial membrane potential (ΔΨm) of cardiomyocytes according to the manufacturer's directions which has been described previously [27]. Briefly, after indicated treatments, cardiomyocytes were loaded with JC-1 for 30 min at 37°C, and images were obtained by using a Leica confocal laser scanning microscope (TCS SP2, Germany). Images of the intensities of the green (JC-1 monomer) and red (JC-1 aggregate) ﬂuorescence intensities were monitored within the individual cells by emission wavelength at 530 nm and 590 nm. Colocalization appeared as an orange red color because of the mixing of the red and yellow signals. Green and red ﬂuorescence intensities were detected using an Infinite™ M200 Microplate Reader. The ΔΨm of cardiomyocytes in each treatment group was calculated as the ﬂuorescence ratio of red to green and was expressed as a multiple of the level in the control groups. As a positive control, mitochondria were depolarized by treating cardiomyocytes with 50 µM carbonylcyanidem-chlorophenyl- hydrazone (CCCP) at 37°C for 5 min. All experiments were repeated at least three times.

### Detection of Cellular ATP Levels

Cellular ATP levels were measured using a firefly luciferase-based ATP assay kit (Beyotime, China) according to the manufacturer's instructions. After the indicated treatments, cardiomyocytes were lysed and centrifuged at 12,000 g for 5 min. Supernatants (100 µL ) were mixed with 100 µL of ATP detection working dilution in a white 96-well plate. Luminance (RLU) was measured by using an Infinite™ M200 Microplate Reader. Standard curves were also generated and the protein concentration of each treatment group was determined using the Bradford Protein assay. Total ATP levels were expressed as nmol/mg protein. This experiment was repeated for three times.

### Detection of Mitochondrial Permeability Transition Pore (mPTP) Opening

The opening mPTP of cardiomyocytes were detected by using the calcein–cobalt with a mPTP assay kit (Genmed Scientifics Inc., USA) according to the manufacturer's directions as described previously [28]. Briefly, cardiomyocytes, seeded in 24-well plates (3×10^5^/per well), were washed with Reagent A, then incubated with Reagents B and C (1∶50; 500 µL per well) at 37°C for 20 min, then washed twice with Reagent A again. Fluorescence intensity was measured using a Infinite™ M200 Microplate Reader (λex 488 nm, λem 505 nm). Cells were subsequently lysed in 20 µL of 0.1 M NaOH and protein concentration was measured using the Bradford Protein assay. The fluorescent signals were normalized to total protein content in the corresponding cell extract. Results were presented as normalized relative ﬂuorescence units (NRFU; U/mg protein).

### Immunocytochemical Analysis of Cytochrome c

After H/R treatment, cardiomyocytes were washed with PBS and were stained for 30 min at 37°C with 250 nM mitochondria-specific dye MitoTrackerRed (Molecular Probes, Cergy Pontoise, France). Then, cardiomyocytes were washed with PBS and were fixed with 4% paraformaldehyde for 20 min and permeabilized with 0.1% Triton X-100 for another 10 min at room temperature. The cells were incubated with 10% normal goat serum for 45 min. Cells were then treated with anti-cytochrome c antibody (1∶100; Abcam, Cambridge, UK) overnight at 4°C. The cells were washed with PBS twice and incubated with Alexa Fluor 488-conjugated secondary antibody (1∶100; Molecular Probes) for 1 h in the dark at 37°C. After washing, the nucleus was counterstained with Hoechst33342 for 10 min. Images of stained cells were obtained by using a Leica confocal laser scanning microscope.

### Measurement of Caspase-3 Activity

As described in detail previously in our experiments [25], [26], [29]. Cardiomyocytes caspase-3 activities were determined by using the caspase-3 Activity Assay kit (Beyotime Institute of Biotechnology, China) according to the manufacturer’s instructions. Results were expressed as the change in enzyme activity relative to the control cultures.

### Statistical Analysis

All the experimental data were expressed as the mean ± SEM. Raw data was analyzed with the GraphPad Prism 5.0 software (GraphPad Software, Inc., SanDiego, CA). Each experiment was carried out at least three times. Statistical analysis for multiple group comparisons was performed by one-way ANOVA, followed by post hoc least significant difference (LSD) tests. All P values were two sided, and *P*<0.05 was considered statistically significant.

## Results

### Effect of Lycopene on the Loss of Cell Viability Induced by H/R

**Figure 1:**
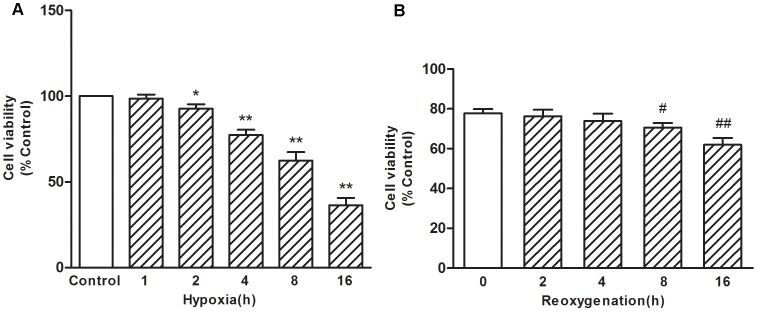
Effects of H/R on cell viability in primary cultured cardiomyocytes. (A) Primary cultured cardiomyocytes were exposed to hypoxia condition for different time (1, 2, 4, 8, 16 h). (B) After 4 h hypoxia, cardiomyocytes were exposed to reoxygenation condition for different time (0, 2, 4, 8, 16 h). After these exposures, cell viability was determined with Trypan blue staining. Data are expressed as percentage of control and represented as mean ± SEM for six independent experiments. *P<0.05, **P<0.01 versus hypoxia 0 h group, #*P<*0.05, ##*P<*0.01 versus reoxygenation 0 h group.

After cardiomyocytes were exposed to various time of hypoxia (1, 2, 4, 8, and 16 h), cell viability was assessed with the Trypan blue staining viability test. We found that cell viability was significantly decreased in a time-dependent manner relative to the control group (Fig. 1A). Exposure to 2 h hypoxia reduced cell viability to 92.7% compared with the control (*P*<0.05), while 4 h, 8 h, and 16 h of hypoxia reduced cell viability to 77.3%, 62.5%, 36.4% of the control respectively (*P*<0.01). The shortest time tested, 1 h, did not induce a significant change in cell viability. In addition, after 4 h hypoxia, the cardiomyocytes underwent reoxygenation for 0, 2, 4, 8, and 16 h respectively. As shown in Figure 1B, cell viability was significantly reduced to 70.6% after 8 h of reoxygenation (*P*<0.05) and 61.9% after 16 h of reoxygenation (*P*<0.01) compared with the 0 h reoxygenation group. Based on these results, treatment with 4 h hypoxia followed by 8 h of reoxygenation was selected for experiments to examine the potential of lycopene-mediated cardiomyocytes protection.

**Figure 2:**
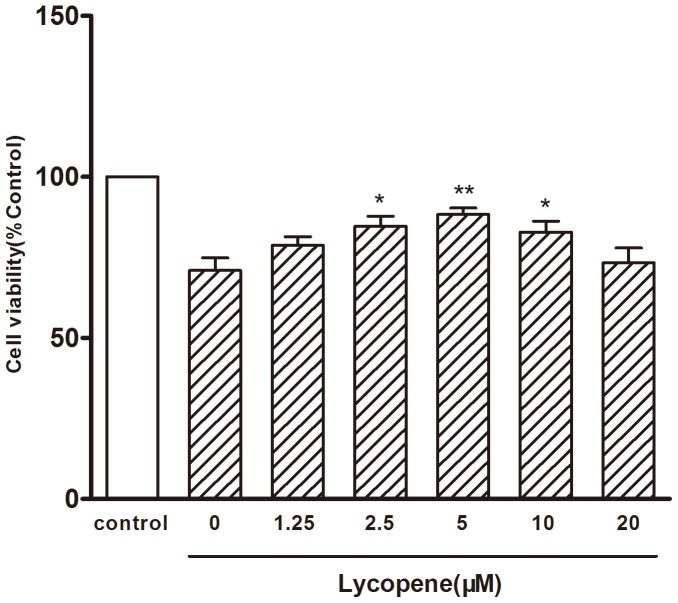
The protective effects of lycopene on H/R-injury. Primary cultured cardiomyocytes were pretreated with lycopene (0, 1.25, 2.5, 5, 10, 20 µM, respectively) for 4 h underwent 4 h hypoxia, then 8 h reoxygenation. *P<0.05, **P<0.01 versus 0 group. Values are mean ± SEM, n = 3.

To investigate the possible cardiomyocytes protective effects of lycopene on H/R injury, cardiomyocytes that were pretreated with lycopene (0, 1.25, 2.5, 5, 10 and 20 µM, respectively) for 4 h underwent H/R treatment. We found that pretreatment with 2.5, 5, and 10 µM lycopene could successfully attenuate the decrease of cell viability caused by H/R treatment (Fig. 2A). The result suggested that lycopene exerted cardiomyocytes protection ability against the H/R-injury in primary cultured mouse cardiomyocytes.

### Lycopene Reduces H/R-induced Apoptosis of Cultured Cardiomyocytes

**Figure 3:**
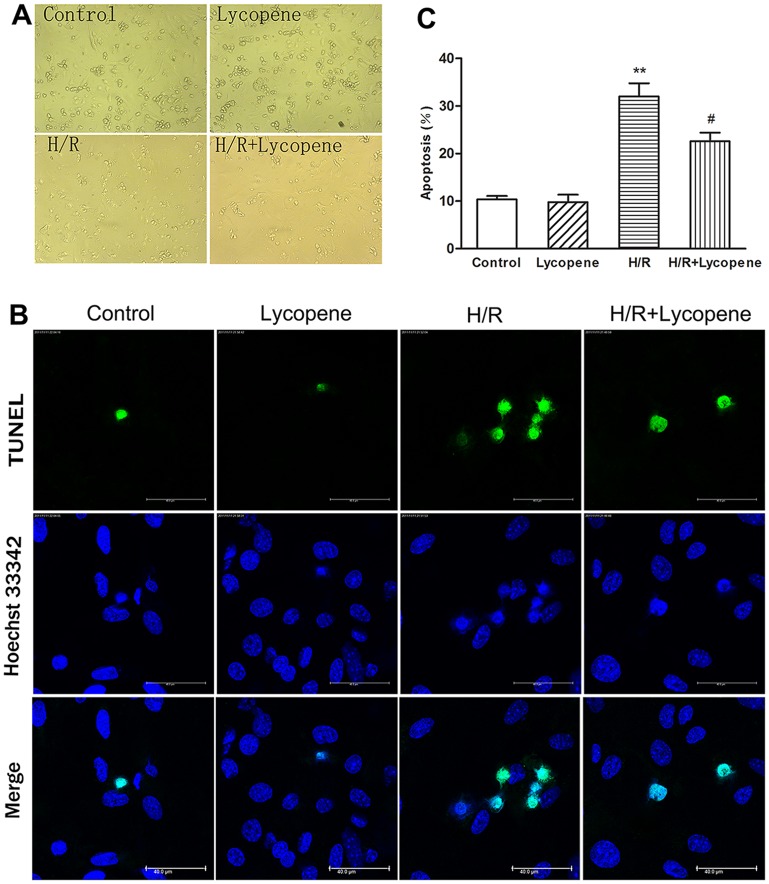
Lycopene protects cultured cardiomyocytes against H/R-induced apoptosis. Cultured cardiomyocytes were treated with 5 µM lycopene for 4 h prior to H/R treatment. (A) Effects of H/R on cardiomyocytes and protection by lycopene. Magnification: 200×. (B) Representative images of TUNEL-positive cells (green, top row) and Hoechst33342 counterstaining (blue, middle row). Scale bar: 40 µm. (C) The histogram shows the relative proportion of TUNEL-positive cells in the different treatment groups. **P<0.01 versus control group, #*P<*0.05 versus H/R group. Values are mean ± SEM, n = 3.

Morphological changes associated with H/R injury were assessed qualitatively by the phase contrast microscopy (Fig. 3A). Many cardiomyocytes were spreading and were simultaneously beating in control cultures. In the H/R-treated cultures, however, cardiomyocytes were rolling up and not beating. These H/R-induced morphological alterations were prevented by the pretreatment with 5 µM lycopene. Then, we investigated the effect of lycopene on the H/R-induced apoptosis in cultured the cardiomyocytes using the TUNEL assay (Fig. 3B and C). More TUNEL-positive cells (green) were observed in H/R-treated cultures (Fig. 3B, top row). Quantitative analysis also showed that H/R induced an increase in TUNEL-positive apoptotic cells compared with cells under regular conditions (*P*<0.01), pretreatment with 5 µM lycopene reduced the amount of TUNEL-positive cells in comparison to the H/R group (*P*<0.05; Fig. 3C).

### Lycopene Reduced the H/R Mediated Oxidative Stress in Primary Cultured Cardiomyocytes

**Figure 4:**
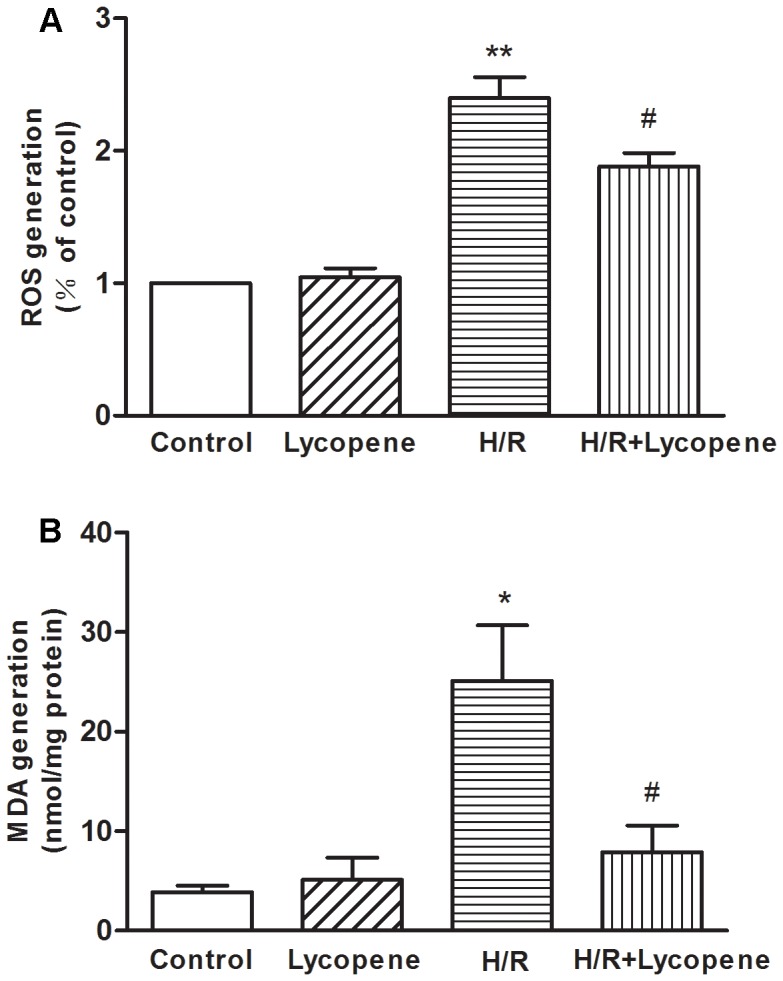
Effects of lycopene on ROS generation and lipid oxidation in cardiomyocytes. Pretreated with lycopene (5 µM) for 4 h, cells were exposed to H/R. (A) Intracellular ROS levels were estimated using the probe DCFH-DA. Fluorescence was read at 485 nm for excitation and 530 nm for emission with a ﬂuorescence microplate reader. Cellular ﬂuorescence intensities were expressed as the multiple of the level in the control groups. (B) Cellular MDA levels were measured using the TBA method, and the concentrations of MDA were expressed as nmol/mg protein. *P<0.05, **P<0.01 versus control group; #*P*<0.05 versus H/R group. Values are mean ± SEM, n = 3.

In the H/R group, exposure to 4 h hypoxia followed by 8 h reoxygenation significantly increased the production of ROS in comparison to that of the control group and lycopene group (*P*<0.01; Fig. 4A). However, the increased amount of ROS in the H/R group could be reduced by pretreatment with lycopene (*P*<0.05; Fig. 4A). These results indicated that H/R had the ability to induce oxidative stress in the cardiomyocytes, however, this effect could be reduced by lycopene pretreatment.

To determine the impact of H/R on oxidative lesions, the intracellular levels of MDA (a common end product of lipid peroxidation) were measured. MDA concentration was elevated significantly in H/R-treated cells (*p*<0.05); however, pretreatment with 5 µM Lycopene decreased H/R-induced MDA generation (*p*<0.05) (Fig. 4B).

### Lycopene Inhibits the Opening of mPTP

**Figure 5:**
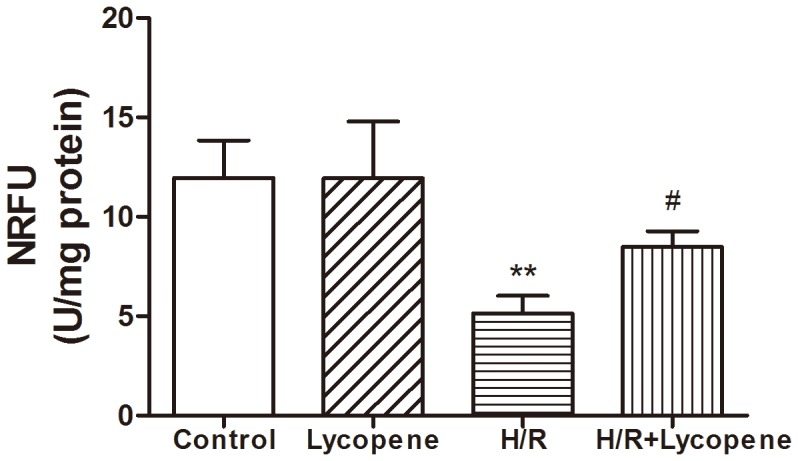
The influence of lycopene on the opening of mPTP in cardiomyocytes exposed to H/R. The mPTP opening was assayed using the calcein–cobalt quenching method. Different group of cardiomyocytes were used to measure the normalized relative fluorescence units (NRFU) of calcein. **P<0.01 compared to the control group, #*P*<0.05 compared to the H/R group. Values are mean ± SEM, n = 3.

Our goal was to test whether the opening of mPTP can be triggered during H/R and whether mPTP inhibition is a mechanism for the protective effects of lycopene. We detected mPTP by using the calcein–cobalt method to monitor the distribution of green fluorescence emitted from calcein as readout of the intact mPTP. A significant decrease in mitochondrial fluorescence of H/R treated cardiomyocytes was observed compared to the control group (*P*<0.01) (Fig. 5). However, lycopene pretreated cultures group demonstrated a significantly higher level of the NRFU compared with the H/R group (*P*<0.05). Taken together our results demonstrate that lycopene has the ability to reduce the extent of opened mPTP in response to H/R treatment.

### Lycopene Prevents Mitochondrial Dysfunction

**Figure 6:**
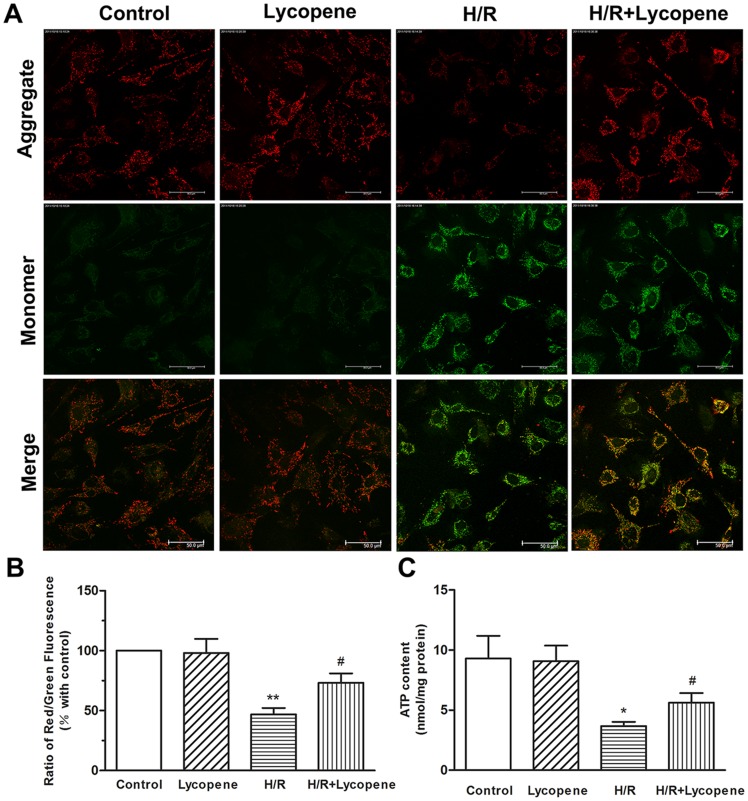
Effects of lycopene on the mitochondrial function in H/R-treated cells. Pretreated with Lycopene (5 µM) for 4 h, cells were exposed to H/R. The treated cells were detected by confocal microscopy and a ﬂuorescence microplate reader. (A) Red fluorescence is from JC-1 aggregates in healthy mitochondria with polarized inner mitochondrial membranes, while green fluorescence is emitted by cytosolic JC-1 monormers and indicates ΔΨm dissipation. Merged images indicate the co-localization of JC-1 aggregates and monomers. Scale bar: 50 µm. (B)The ΔΨm of the cardiomyocytes in each group was calculated as the ﬂuorescence ratio of red to green, and they were expressed as the multiple of the level in control groups. (C) Effects of lycopene on the ATP content in H/R-treated cells. ATP concentrations were determined using an ATP Determination Kit, and the ATP levels were expressed as nmol/mg protein. *P<0.05, **P<0.01 versus control group; #*P*<0.05 versus H/R group. Values are mean ± SEM, n = 3.

To further investigate the effects of lycopene on the mitochondrial function, indicators of mitochondrial activity such as membrane potential (ΔΨm) and levels of cellular ATP were determined. We estimated ΔΨm by using the JC-1 probe. The JC-1 probe detected both red polarized mitochondria and green depolarized mitochondria in the control cells, which represented the ΔΨm under physiological conditions (Fig. 6A). H/R treated cardiomyocytes showed a decrease in the red fluorescence and a significant increase in the green fluorescence, and lycopene pretreatment reversed the changes in fluorescence. The fluorescence ratio of red to green quantified the ΔΨm, and a low ratio represented mitochondrial depolarization. We also found that the ratio of H/R group was lower than control (*P*<0.01) and lycopene pretreatment showed the protective effects compared to the H/R-treated groups (*P*<0.05; Fig. 6B). Cellular ATP content was also a sensitive readout of mitochondrial function. As shown in Fig. 6C, ATP concentrations significantly decreased from 9.30 nmol/mg protein in the control group to 3.67 nmol/mg protein in the H/R group (*p*<0.05). In contrast, pretreatment of cultures with lycopene resulted in an increase of cellular ATP level compared with H/R group (*P*<0.05). These findings provided evidences that lycopene protected mitochondrial function during H/R.

### Lycopene Blocks the Mitochondrial Apoptotic Pathway

**Figure 7:**
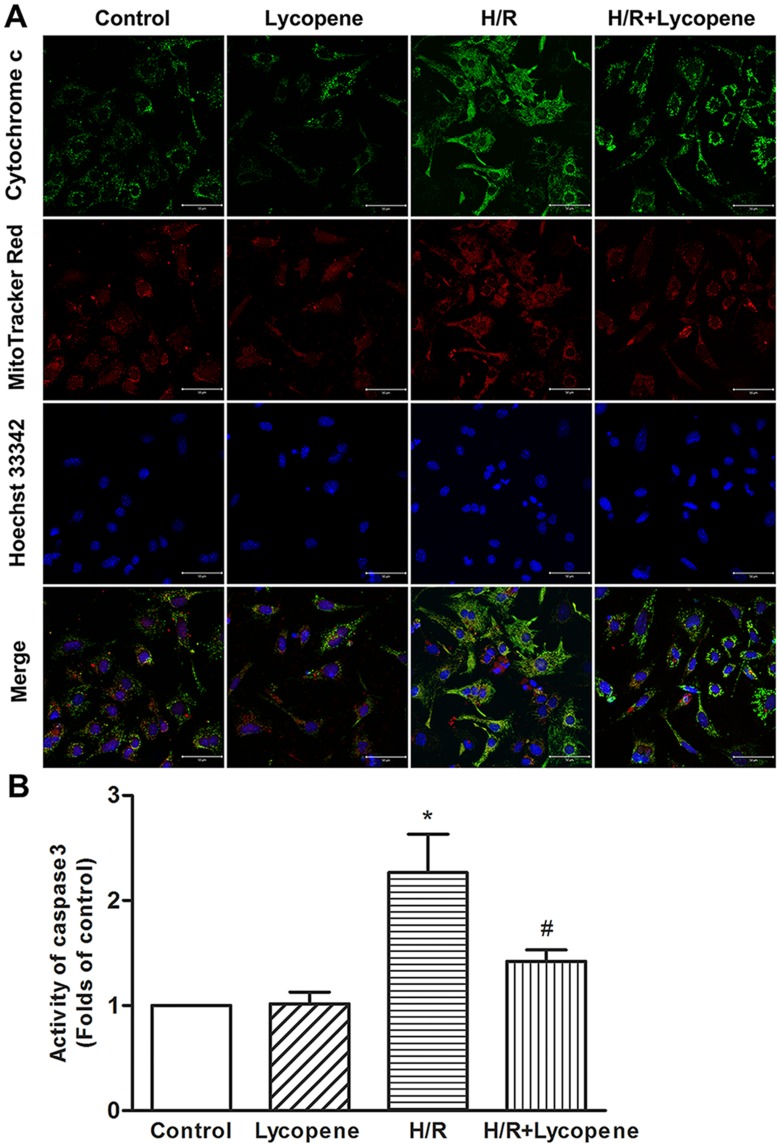
Lycopene inhibited H/R-induced cytochrome c release and caspase-3 activation in cardiomyocytes. (A) Lycopene inhibited H/R-induced cytochrome c release. Pretreated with lycopene (5 µM) for 4 h, cells were exposed to H/R. The nucleus was stained with Hoechst33342. Mitochondrial cytochrome c release was detected by colocalization of MitoTracker Red and cytochrome c (green) immunocytochemical signals under confocal microscopy. Scale bar: 50 µm. Images presented are representative of three independent experiments. (B) Lycopene inhibited H/R-induced caspase-3 activation. Cardiomyocytes were treated as described in (A). Caspase-3 activity was detected using a commercial kit as described in Methods. Data are presented as fold-change from the control group. *P<0.01 versus control group. #*P*<0.05 versus H/R group. Values are mean ± SEM, n = 3.

Mitochondrial dysfunction may initiate apoptosis by releasing pro-apoptotic factors, such as cytochrome c, from the mitochondrial intermembrane space into the cytoplasm to trigger apoptosis via a caspase-3-dependent pathway [30], [31]. Accordingly, changes in mitochondrial cytochrome c were measured by simultaneous the cytochrome c immunoﬂuorescence and MitoTracker Red ﬂuorescence. As shown in Figure 7A, H/R treatment decreased colocalization of cytochrome c and MitoTracker Red (peripheral green ﬂuorescence, bottom row) which indicated the release of cytochrome c from the mitochondrial matrix into the cytosol. This subcellular shift in cytochrome c was markedly inhibited by 5 µM lycopene pretreatment.

To further investigate the effects of Lycopene on caspase-3 activation, we measured caspase-3 activity (Figure 7B) by a spectrophotometric assay in cell lysates from four different groups. Cardiomyocytes with H/R treatment showed a significant increase in caspase-3 activity (*P*<0.05). In contrast, pretreatment with 5 µM lycopene significantly inhibited the H/R-induced activation of caspase-3 (*P*<0.05), suggesting that the lycopene reduced H/R-induced apoptosis was mediated through the blockage of caspase-3-dependent cardiomyocytes apoptosis.

## Discussion

In the present study, by using the primary cultured cardiomyocytes, we confirmed that apoptosis was important in H/R-injury [12]. Furthermore, the present result demonstrated that H/R-induced apoptosis was closely associated with mitochondrial dysfunction. Importantly, the H/R-induced apoptosis was efficiently prevented by lycopene pretreatment. To our knowledge, this is the first report to elucidate the cardiomyocytes protective action of lycopene in cardiomyocytes in response to the H/R-injury.

In the present study, the possible correlation between lycopene and mitochondria function was evaluated. It is well known that mitochondria are the key components to the function of cardiomyocytes [32] and that mitochondrial dysfunction is the primary cause of H/R-induced apoptosis of cardiomyocytes [17]. A critical factor mediating mitochondrial dysfunction is the opening of mPTP. Upon activation of mPTP, functional breakdown and morphological disintegration of mitochondria occurs and thus initiates cell death [13]. In this study, we further confirmed that the anti-apoptotic action of lycopene was mediated by suppressing the ROS dependent mPTP opening to prevent mitochondrial dysfunction and blocked the activation of the cytochrome c/caspase-3 apoptotic pathway.

The increased production of ROS mainly formed in the mitochondrial respiratory chain could result in an oxidative damage to mitochondrial proteins and DNA are highly relevant to myocardial failure and atherosclerosis [33]. Moreover, ROS can cause thiol oxidation and inhibition of the ATP synthesis and adenine nucleotide translocate (ANT). They can also cause peroxidation of the unsaturated fatty acids in membrane phospholipids, especially cardiolipin at the inner mitochondrial membrane, which leads to further inhibition of respiratory chain activity [34], [35]. As a consequence of lipid peroxidation, reactive aldehydes such as 4-hydroxynonenal are released and can modify membrane proteins [36]. In this study, we found that ROS levels and lipid oxidation increased in H/R-treated cardiomyocytes, and were effectively attenuated by lycopene. The anti-oxidative potential of lycopene should be accounted for its ability to preserve the activity of endogenous free radical scavengers, and protect respiratory chain complexes directly [37], which prevents ROS production and secondary oxidative damage.

It has been shown that an increase in intracellular ROS predisposed cells to damage [3], [38] and involved numerous signaling pathways. Initiation of ROS-dependent mPTP opening has specifically been implicated in cardiomyocytes death [39]. The mPTP is located at the junction between the internal and outer membranes of the mitochondria (intermembrane compartment/space). The diameter of a mPTP allows molecules up to ∼1500 Da to cross the mitochondrial membrane. During H/R, the elevated cellular ROS levels, combined with the excessive amounts of Ca^2+^ and adenine nucleotide depletion, trigger the opening of mPTP [40]. Given the anti-oxidative ability, we hypothesize that there was a role for lycopene in the regulation of mPTP. This hypothesis was supported by the observation that lycopene was able to partially inhibit the opening of mPTP and decrease ROS levels in cardiomyocytes.

The opening of the mPTP can lead to a bioenergetic, biosynthetic, and redox crisis in a cell that can directly threaten the survival of the cell [41]. When the mPTP is open, the mitochondrial inner membrane becomes permeable to protons which then lead to the uncoupling of the electron respiratory chain and the collapse of membrane potential, which in turn leads to a cessation of ATP generation in mitochondria [41], [42]. In the pretreated cardiomyocytes with lycopene, the H/R-induced loss of mitochondrial membrane potential and decline in ATP levels were alleviated, supporting the notion that the protection of cardiomyocytes is, in part, due to the prevention of mitochondrial dysfunction. These results were consistent with recent studies that had demonstrated the ability of lycopene to enhance the mitochondrial activity of HepG2 cells exposed to aflatoxin B1 [43] and to improve the mitochondrial function in nervous system of rats exposed to 3-nitropropionic acid [37].

Another threatening consequence of the altered mitochondrial permeability is the release of apoptogenic proteins from the mitochondrial intermembrane space into the cytosol [41], [42]. Cytochrome c is associated with the inner mitochondrial membrane and serves as an essential component of the electron transfer chain. With opening of the mPTP and translocation of cytochrome c into the cytosol, mitochondrial function is compromised. In this study, the release of cytochrome c from the mitochondrial matrix into the cytosol was significantly inhibited by the 5 µM lycopene pretreatment; the results of this study demonstrated a correlation exists between lycopene and the preservation of mitochondrial function. Moreover, once the cytochrome c enters the cytosol, it becomes associated with the apoptotic peptidase activating factor 1 (Apaf-1) and dATP in order to form an apoptosome. Apoptosomes recruit and activate procaspase-9, which in turn activates other downstream caspases to initiate apoptosis [42]. Caspase-3 has classically been considered as an executioner caspase. In this experiment, lycopene suppressed the activation of caspase-3 and was associated with lowering the cytosolic levels of cytochrome c. In addition, recent studies have revealed that lycopene participates in the intrinsic apoptosis process by inhibiting the mitochondrial apoptotic pathway [28]. Although the underlying mechanisms required further study, the present study demonstrated that lycopene inhibits the intrinsic signaling pathways of apoptosis.

In conclusion, mitochondrial dysfunction may be the underlying cause of the H/R-induced apoptosis in mouse cardiomyocytes, whereas lycopene may provide protective benefit by maintaining the mitochondrial function. Interactions between lycopene and the mitochondria provided a new strategy to protect against the H/R-induced apoptosis by improving the mitochondrial function. Future experiments are required to evaluate the protective efficacy of lycopene in vivo, and the possible molecular mechanism will be clarified.
